# Stem Cell Therapy: A New Treatment for Burns?

**DOI:** 10.3390/ph4101355

**Published:** 2011-10-21

**Authors:** Anna Arno, Alexandra H. Smith, Patrick H. Blit, Mohammed Al Shehab, Gerd G. Gauglitz, Marc G. Jeschke

**Affiliations:** 1 Ross Tilley Burn Centre, Sunnybrook Health Sciences Centre, University of Toronto, 2075 Bayview Avenue, Toronto, Ontario M4N 3M5, Canada; 2 Plastic Surgery Department and Burn Unit, Vall d'Hebron University Hospital, Autonomous University of Barcelona, Passeig de la Vall d'Hebron 119-129, 08035, Barcelona, Spain; 3 Department of Dermatology and Allergology, Ludwig Maximilians University, Geschwister-Scholl-Platz 1, 80539, Munich, Germany

**Keywords:** stem cells, burn, tissue engineering, regenerative medicine

## Abstract

Stem cell therapy has emerged as a promising new approach in almost every medicine specialty. This vast, heterogeneous family of cells are now both naturally (embryonic and adult stem cells) or artificially obtained (induced pluripotent stem cells or iPSCs) and their fates have become increasingly controllable, thanks to ongoing research in this passionate new field. We are at the beginning of a new era in medicine, with multiple applications for stem cell therapy, not only as a monotherapy, but also as an adjunct to other strategies, such as organ transplantation or standard drug treatment. Regrettably, serious preclinical concerns remain and differentiation, cell fusion, senescence and signalling crosstalk with growth factors and biomaterials are still challenges for this promising multidisciplinary therapeutic modality. Severe burns have several indications for stem cell therapy, including enhancement of wound healing, replacement of damaged skin and perfect skin regeneration – incorporating skin appendages and reduced fibrosis –, as well as systemic effects, such as inflammation, hypermetabolism and immunosuppression. The aim of this review is to describe well established characteristics of stem cells and to delineate new advances in the stem cell field, in the context of burn injury and wound healing.

## 1. Introduction

Burn injury is a devastating trauma with systemic consequences. Although survival rates are increasing, burn injury remains a great challenge in the field of cutaneous wound healing. Major burn patients lack enough skin to cover their burns and the currently used cutaneous substitutes and cultured epithelial autografts (CEA) are still neither efficient nor effective solutions [1,2]. Transplanted skin from donors is currently not an option due to rejection; however, augmenting immunotolerance via stem cell therapy may overcome this problem. Regenerative medicine using stem cells (SC) is an efficient, low-morbidity and high-quality therapy for skin coverage in burns, mainly due to the regeneration of skin appendages [3] and the minimal risk of hypertrophic scarring [4]. Furthermore, stem cells may be able to address the other systemic effects of burn injury, such as hypermetabolism and inflammation [5]. Further research is needed to analyze long-term effects of SCs and unravel the optimal method of administration (when and with which matrix).

## 2. Skin Regeneration

Skin maintains homeostasis by temperature regulation via hair follicles, sweat glands and dermal capillaries, and by lubrication via sebaceous glands. Burn injury represents a cellular stress in the skin. Normal adult skin repair is slow, with high risk of infection and hypertrophic scarring [3]. Epidermal keratinocytes form a scar without cutaneous appendages, such as hair follicles, sweat or sebaceous glands (Table 1). The regenerated epidermis is thin and has fewer and flatter epidermal ridges.

Stem cells exist in adult tissue on reserve to repair cells following stress signals [6]. The vast majority of skin stem cells are located in the hair follicle bulge [7]. To repair and reepithelialize a wound, stem cells from the hair follicle bulge give rise to daughter skin cells, which migrate to the epidermis (basal layer and sebaceous gland) [8] (Table 2). In adult skin, superficial burns that leave hair follicles intact are healed rapidly with the regeneration of epidermal appendages. Deeper injuries that affect the hair follicle bulge heal with a scar and without adnexal structures [3].

Regenerative medicine aims to not only accelerate reepithelialization after burn injury, but also to reconstruct functional skin with sweat glands, hair follicles and dermal capillaries. These goals might be achieved by stem cell therapy. Approaches to stem cell therapy include local recruitment of endogenous SCs or SC transplantation (often *in vitro* modified), either of which can be combined with gene therapy or tissue engineering. Tissue engineering is an experimental procedure that combines cellular biology, engineering and medicine to develop three-dimensional tissues and restore function [9].

## 3. Stem Cells Definition and Classification

### 3.1. Stem Cells Definition

Stem cells (SCs) are defined by two main characteristics: their capacity of prolonged self-renewal (proliferation) and multilineage differentiation (asymmetric replication) [3,10]. These characteristics are more pronounced in younger sources [11]. By asymmetric replication, after every cell division, one cell retains its self-renewing capacity, while the other (transit-amplifying or TA cell) enters a differentiation pathway and joins a mature non-dividing population [12]. When an unspecialized stem cell differentiates, it assumes characteristics of a specific tissue [13]. SCs are pluri-, multi- or unipotent [14]. The zygote is the only mammalian cell capable of producing all cells and tissues of an organism and thus is considered totipotent [15]. Pluripotent cells produce cells and tissues belonging to all three germ layers—Ectoderm, mesoderm and endoderm [16]. Multipotent cells produce more than one cell lineage, within a closely related family of cells. Unipotent cells only differentiate into a single cell phenotype [17]. Plasticity describes the phenomenon whereby SCs from one tissue produce cell types of a completely different tissue [18]. SCs can remain undifferentiated, in which state there is risk of uncontrolled proliferation and tumor formation [11]. SCs have a slow-cycling nature *in vivo*, high proliferative potential and participate in tissue regeneration and repair, during both fetal development and adult wound healing [19].

### 3.2. Stem Cells Classification

When classified by origin, there are two types of stem cells: embryonic (ESC) and non-embryonic stem cells. The latter are also referred to as adult (ASC) or somatic stem cells (however, SCs derived from the placenta or umbilical cord are also considered ASCs) [20,21]. Embryonic germ cells are derived from the primitive gonadal ridges of the developing embryo or fetus (6-9 weeks gestation in the humans) and have many of the pluripotential properties of ESCs [22].

#### 3.2.1. Embryonic Stem Cells (ESCs)

ESCs are pluripotent stem cells derived from the inner cell mass of an early stage embryo known as blastocyst [10] and give rise to all cells of the three embryonic germ layers: endoderm, mesoderm and ectoderm. Human Embryonic Stem Cells (hESCs) are derived from excess developing pre-implantation embryos (5 day-old embryos, 4-8 day-old morula, or inner cell mass of blastocysts) that have usually been fertilized *in vitro* at a fertilization clinic. ESCs can be propagated indefinitely in an undifferentiated state [23] *in vitro* using either feeder layers or extracellular stimuli (e.g., cytokines or growth factors) [24]. Derivation of human embryonic cell lines is controversial because it requires destruction of an embryo [11], may develop teratocarcinomas (tumours composed of tissues from all three germ layers [25]), are immunogenic and show genetic instability *in vitro* [15]. Accordingly, adult stem cell research is currently favoured [6,26,27]. Apart from being used in regenerative medicine, ESCs may be used to perform developmental, genetic (through knock-out technology) and pharmacological research. hESC-based *in vitro* studies of drug toxicity have proven to be an accurate alternative to experimental animal models [28].

ESCs are capable of unlimited expansion *in vitro* and are considered an immortal epiblast derivative with a checkpoint in differentiation that enables their expansion as undifferentiated colonies, which can be instructed to maintain this phenotype indefinitely [23].

#### 3.2.2. Adult Stem Cells (ASCs)

SC clinical studies have increased during the past two decades in almost every field of medicine; including, haemato/immunotherapies [29-32], diabetes mellitus [33], chronic degenerative illnesses (e.g., in the field of rheumatology) [34-37], cardiovascular diseases [38], multiple sclerosis and other neuropathies [39], corneal repair [40] and wound healing [41].

Adult stem cells (ASCs) were discovered in the mid-1950s; they are found in low abundance in almost all adult tissues and in high abundance in the umbilical cord [10]. They are found in special regulatory niches as self-renewing progenitor cells that are able to produce one or more specialized cell types. ASCs are usually considered to be tissue specific, self-renewing populations of cells, which can differentiate into cell types associated with the organ system in which they reside [42].

Slowly replicating and bromodeoxyuridine-label-retaining, ASCs are under strict regulatory control of their mobilization and differentiation [23]. ASCs are less potent (usually only uni- or multipotent) and have less differentiation potential than ESCs. Distinct from ESCs, ASCs are not capable of unlimited expansion *in vitro*. ASC potency and plasticity is still in contention, though [11,22,43].

ASCs include mesenchymal stem cells (MSCs), hematopoietic stem cells (HSCs), epithelial and neural stem cells, and others. HSCs and MSCs originate in the bone marrow (MSCs may have additional origins, as we will describe later) and differentiate into endothelium, liver, bone, muscle, skin or others [11].

Epidermal and dermal SCs are also multipotent. It has been suggested that they not only contribute to skin production, but can also be stimulated into neural and osteogenic lineages [44,45].

#### 3.2.3. Induced Pluripotent Stem Cells (iPSCs)

**Figure 1:**
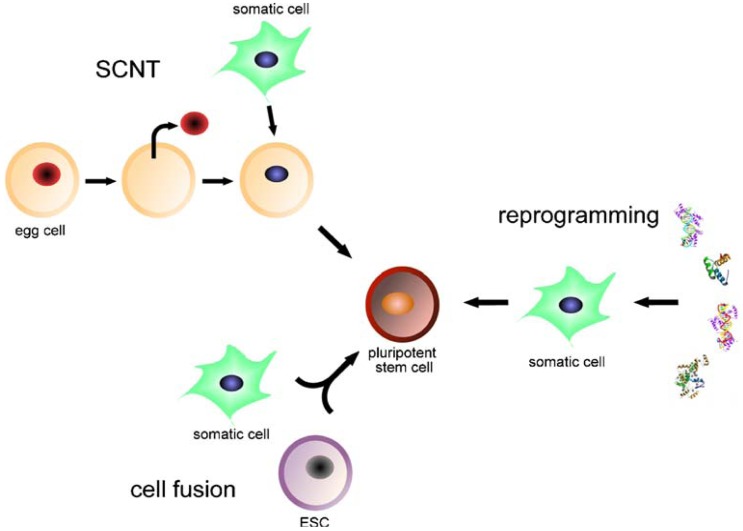
Methods of production of induced pluripotent stem cells. Reproduced from [10] with permission from Rightslink.

Induced pluripotent stem cells (iPSCs) are artificially derived from non-pluripotent cells, typically adult somatic cells (mostly fibroblasts of murine or human origin), most frequently by epigenetic reprogramming and also by nuclear transfer or cell division [46]. Expression of transcription factors characteristic for undifferentiated embryonic stem cells is induced; for example, OCT4 (also known as POU5F1, being the most important one), SOX2, c-MYC, KLF4, Lin28 and/or NANOG [6,10,15,47,48] (Figure 1). Transcription factors or cell markers are the key mediators of cellular identity [49]. Direct reprogramming (also referred to as transdifferentiation) describes ectopic expression of defined transcription factors, a very slow and inefficient process that may limit the quality of resulting iPSCs [50]. For example, fibroblasts can be reprogrammed into neurons, cardiomyocytes and blood-cell progenitors [51-53]. Small molecules may improve the reprogramming efficiency but increase its tumorigenicity [54]. Elements that influence reprogramming include the respective donor cell type, the transcription or reprogramming factors utilized, the mode of delivery (e.g., virus, RNA, *etc.*) and the culture conditions, all of which depend on the purpose of the process [46].

Broadly, we could imagine iPSCs as “artificial ESCs”. iPSCs represent stable lines of embryonic-like pluripotent stem cells [55]. In contrast to ESCs, human iPSCs can be derived from the patient to be treated (for autologous cell therapy), reducing the risk of HLA mismatching and immune rejection [56]. iPSCs may also be used for establishing *in vitro* disease models, drug or toxicity screening, and basic gene research [46]. iPSCs represent a widely available, non-controversial, non-restricted and practically infinite source of pluripotent cells. Nonetheless, they still share with classic ESCs the critical disadvantage of malignancy transformation [10]; although, integrative delivery systems with consequent deletion seems to lower the risks of iPSCs oncogenesis [54]. A multitude of protocols for iPSCs generation have been developed in recent years, spanning across different mouse and human donor populations and varying in the number, identity and delivery of the reprogramming factors [57-60].

## 4. Adult Mesenchymal Stem Cells

Ideally, stem cells for regenerative medicine should be abundantly available, accessible by a minimally invasive procedure and then safely and effectively transplanted to either an autologous or allogeneic host [6]. As previously mentioned, tumorigenicity and ethical considerations have impeded the widespread clinical use of ESCs [11]. Instead, most regenerative medicine research is focused on iPSCs and ASCs; in particular, adult mesenchymal stem cells (MSCs) [61,62].

MSCs are derived mainly from bone marrow and adipose tissue [63,64], and to a lesser extent, placenta [65], amniotic fluid [61], umbilical cord [66], dental pulp [67], tendon [68], trabecular bone [69] and synovia [70]. Actually, MSCs may reside in all post-natal tissues. Bone marrow (where they were first identified) and adipose tissue are the main sources of MSCs for cell therapy, due to high expansion potential and reproducible isolation protocols [71]. CD34+ hematopoietic stem cells are the most widely studied and represent the only currently clinically approved stem cell [12,71].

Human mesenchymal stem cells (hMSCs) are characterized by three criteria: (1) plastic-adherent under culture; (2) capacity to differentiate into at least three mesenchymal lineages: bone, fat and cartilage; (3) express cell markers CD73, CD90, CD105 and negative for CD11b, CD14, CD34, CD45 and HLA-DR [62,72].

MSCs release various cytokines and growth factors that influence the microenvironment by either modulating the host immune response or stimulating resident cells [73], conferring anti-fibrotic, anti-apoptotic, pro-angiogenic and immunosuppressive properties [74,75]. Mediators involved in MSC-mediated immunomodulation include interferon-γ, toll like receptors, tumor necrosis factor-α, interleukin (IL)-1α, IL-1β, indoleamine 2,3-dioxygenase, leukemia inhibitory factor, HLAG5, IL-10, transforming growth factor (TGF)-β1, hepatocyte growth factor, heme oxygenase1, IL6, IL-1 receptor antagonist (IL-1RA) and prostaglandin E_2_ [71,75-80]. MSCs also stimulate the proliferation of other progenitor cell populations within target organs to promote endogenous repair [77].

MSCs have a great potential in tissue engineering and may serve to treat chronic inflammatory and degenerative disorders due to their immunosuppressive properties [75,81]. They are currently being tested in several clinical trials for osteoarthritis, osteogenesis imperfecta, articular cartilage defects, osteonecrosis and bone fracture [82,83]. They are considered ‘immunoprivileged’ by many researchers and may permit allo-transplantation without immunosuppressive therapy, which would become particularly useful in treating acute injuries [84,85]. Having said that, animal studies have shown that intramyocardial injection of MSCs may differentiate into encapsulated structures with calcifications and ossifications, raising the possibility of malignant transformation [77].

## 5. Main Sources of Adult Stem Cells

### 5.1. Bone Marrow-Derived Stem Cells (BMSCS)

Bone marrow has been the primary source of mesenchymal stem cells; however, bone marrow collection is invasive and MSC isolation is inefficient (<0.05%) [86,87]. BMSCs are able to undergo cell fusion, a natural process of mingling of genetic material that modifies gene expression patterns [88]. Cell fusion is implicated in regeneration, normal development, immune response and tissue formation and plays a prominent role in stem cell plasticity [89]. Indeed, cell fusion can modify the gene program and govern cell fate, transforming the cell into a more immature state to achieve a regenerative function [90].

Haematopoietic stem cell transplantation is the first and most widely available stem cell therapy [91]. hMSCs derived from bone marrow are capable of differentiating into epithelial cells of the liver, lung, gastrointestinal tract and skin [92].

Autologous bone-marrow derived cultured hMSCs have been applied to wounds, using a specialized fibrin spray system; this approach is currently being performed and supposed to be safe and valid for topical cell administration, although with no solid data to support its validity [93].

### 5.2. Adipose Tissue-Derived Stem Cells (ADSCS)

Adipose-derived stem/stromal cells (ADSCs) are multipotent somatic stem cells that can differentiate into several lineages, including adipose cells, chondrocytes, osteoblasts, endothelial cells, epithelium, cardiomyocytes and neuronal cells [94]. The existence of stem cells within adipose tissue was reported for the first time in 2001 [95]. They are often described as processed lipoaspirate cells (PLA), preadipocytes, or adipose stem cells, although the international Fat Applied Technology Society recommends the term “ADSCs” [6]. ADSCs express mesenchymal cell-specific markers and molecular markers typical for the embryonic stem cell phenotype: OCT4, Nanog and Sox2 [96,97]. ADSCs are heterogeneous, differing depending on their anatomical regions and depending on their type (white or brown) [98].

Current research suggests that they may actually be pluripotent and form cell types of all three germ layers [99]. ADSCs represent a promising source of adult mesenchymal stem cells, mainly because isolation is less invasive and more efficient [6,71]. Aspirated fat is in plentiful supply in many plastic surgery procedures—e.g., liposuction and liposculpture—and precursor cells can be purified to obtain the ADSC-rich stromal vascular fraction (SVF) [100]. The SVF is a heterogeneous mixture containing endothelial cells, preadipocytes, fibroblasts, vascular cells, macrophages, and numerous mesenchymal stem cells [6] and is now studied as a supplement to free fat transfer in order to increase yield [101]. Expansion of ADSC populations in culture can yield 100 to 1,000 times more progenitor cells than isolation from bone marrow [102].

Besides being one of the richest sources of ASCs in the human body, adipose tissue is also an endocrine organ that secretes numerous hormones, growth factors and cytokines, that support wound healing and other functions such as leptin, epidermal growth factor, tumor necrosis factor-α, basic fibroblast growth factor, keratinocyte growth factor, transforming growth factor-β1 (TGF-β1), vascular endothelial growth factor, hepatocyte growth factor, interleukin (IL)-6, IL-7, IL-8, IL-11, IL-12, macrophage-colony stimulation factor, platelet-derived growth factor, brain-derived neurotrophic factor, granulocyte colony stimulating factor and leukaemia inhibitory factor [6,102]. These paracrine influences are of great importance in many stem cell therapies, creating a favourable environment for development of more functional cells and tissue repair, through promotion of neovascularization, endogene repair mechanisms and regulation of immune responses [77,103].

ADSCs represent a highly efficient source of iPSCs [104]. They may be used to test drug toxicity, reducing the need for animals. They may also offer an important tool for cell-based gene therapy in the field of wound healing, because ADSCs can efficiently (above 60%) be transducted with vectors [12,105].

Various clinical trials have shown the regenerative capability of adipose-derived stem cells in subspecialties of medical fields such as plastic surgery (for breast reconstruction and aesthetic lipofilling), general surgery (to heal chronic fistulas in Crohn's disease), orthopedic surgery, oral and maxillofacial surgery (to stimulate bone repair in calvarial defects) and cardiology (ischemic heart disease and acute myocardial infarct) [77,106,107]. With the risk of malignancy transformation and heterogeneity, however, there is still not enough evidence to encourage wide and safe use of ADSCs and further research is required [108-110]. Future studies to optimize the differentiation of ADSCs into deficit cells may unlock their therapeutic potential in regenerative medicine.

All in all, ADSCs hold great promise for use in tissue repair and regeneration, due to their availability, pro-angiogenic an anti-apoptotic factor secretion, immunomodulatory effects and multilineage differentiation, becoming one of the most popular adult stem cells currently explored [6,77,100,103,111].

### 5.3. Umbilical Cord (Blood) Derived Stem Cells

Umbilical cord (UC) and cord blood-derived stem cells remain the world's largest potential source of stem cells, considering the global birth rate of around 135 million per year [86]. The umbilical cord represents a well known source of endothelial progenitor cells [112]. Umbilical cord blood contains haematopoietic as well as non-haematopoietic stem cells, these latter also named as CBEs (Cord Blood Embryonic-like stem cells) [113,114]. CBEs have been shown to differentiate into neural, hepatobiliary, pancreatic-like precursors and potentially others [115,116].

Human umbilical cord blood is a rich source of hemopoietic stem cells for clinical application and may be one of the largest sources of stem cells with naive immune status [12,101].

**Figure 2:**
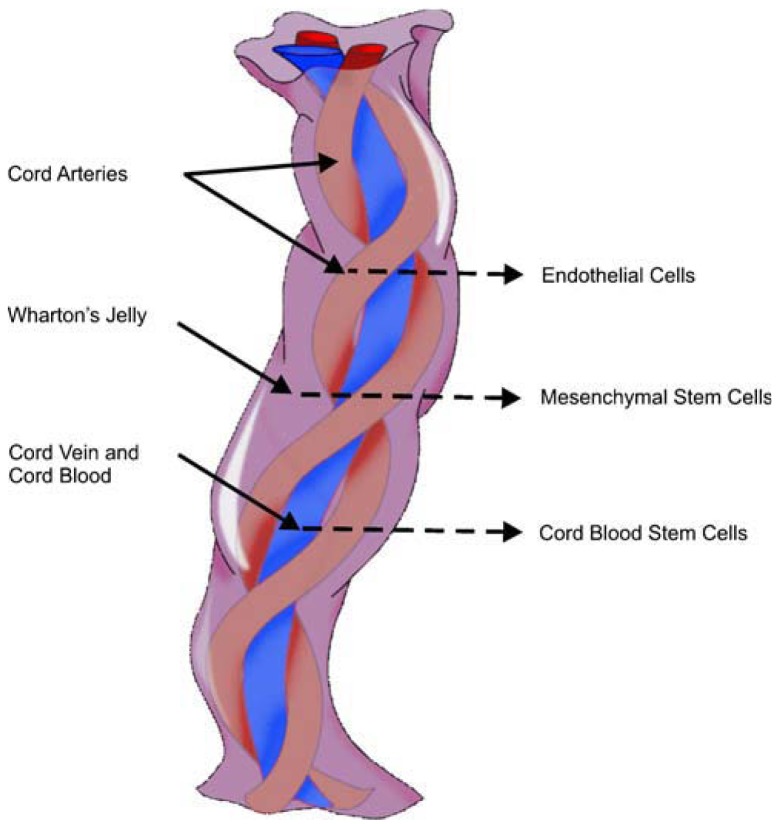
The anatomy of the umbilical cord. Reproduced from [10] with permission from Rightslink.

From innermost to outermost, the layers within the UC include the vessels, Wharton's jelly or matrix, and amniotic membrane or cord-lining, epithelium or subamnion [86]. Wharton's jelly gives rise to MSCs and has lower cell density, yet allows us to quickly isolate many cells [117]. A single piece of 5–10 mm^3^ of Wharton's jelly has the potential to yield up to 1 billion MSCs in 30 days; bearing in mind that the average UC measures 50 cm [15], it undoubtedly represents a rich source of SCs. Human umbilical cord perivascular cells are nearly identical to Wharton's jelly MSCs [118] (Figure 2).

Amniotic membrane and cord-lining are sometimes interchangeable words referring to the UC membrane in general, or referring to different cell types. The outer membrane of the UC is an extremely rich source of stem cells for burn resurfacing [12]. The cord lining gives rise to multipotent epithelial stem cells (CL-epithelial stem cells) [86].

**Figure 3:**
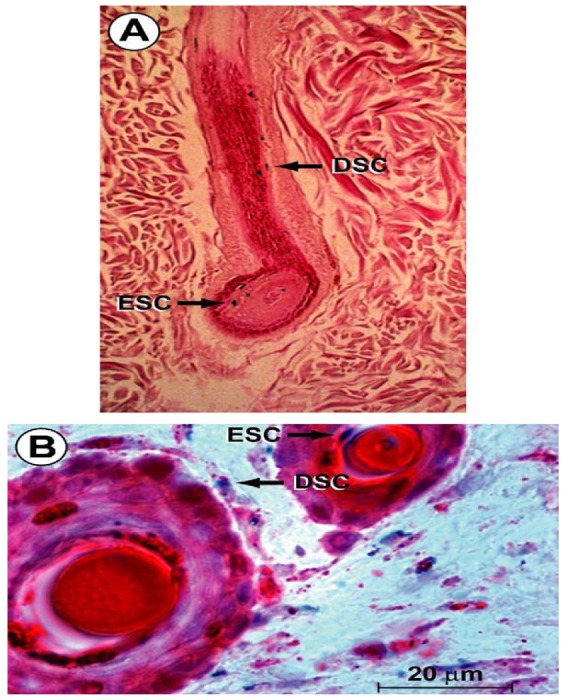
Photomicrographs of cord lining-epithelial cells (A) and cord lining-mesenchymal cells (B). Reproduced from [12] with permission from Righstlink.

Cord lining-mesenchymal stem cells (CL-MSC) express CD23, CD14 and low amounts of CD34 and CD35; they do not express endothelial marker CD31 and have greater *in vitro* expansion than Wharton's jelly-derived MSCs [119]. CD14 inhibits T cells. Wharton's jelly derived MSCs do not express CD14 or CD23. Despite those descriptions, the cell markers of umbilical cord-derived MSCs are under great debate [86,120] (Figure 3).

Generally, umbilical cord-derived MSCs can differentiate into bone, skin, endothelium, hepatocyte, neural lineages and others. Amniotic membrane-derived MSCs specifically can differentiate into bone, cartilage and fat [12,121].

Regarding hematologic diseases, the immaturity of umbilical cord blood (UCB) cells is associated with low immunogenicity, which reduces their graft-versus-host reactivity compared to adult-derived bone marrow grafts [122]. On the other hand, umbilical cord blood supplies multipotent stem cells at a rate 30% lower than that achieved from adult bone marrow [101]. Umbilical cord blood was introduced as an alternative source of allogeneic HSCs after the success of cord transplantation in a child with Fanconi's anemia. Both cord blood transplants and matched unrelated bone marrow transplants share similar disease-free survival and transplant-related mortality [91]. Further research will need to clarify when allogeneic cord blood transplantation is best indicated [124].

With respect to burns and skin wound healing, umbilical cord and amniotic membrane may emerge as new promising sources of “off-the-shelf” cell-engineered skin [86]. Moreover, co-administration of several types of stem cells may elicit synergistic benefits [77], suggesting the use of both epithelial and mesenchymal stem cells.

### 5.4. Hair-Follicle Stem Cells

Hair follicles are a promising source of easily accessible multi (or pluri) potent stem cells which are non-oncogenic and carry no ethical concerns, in contrast to embryonic stem cells or induced pluripotent stem cells. In fact, many researchers consider hair follicles to be the most promising source of multipotent stem cells [19,124].

Hair follicle pluripotent stem cells of the scalp are positive for nestin and the embryonic stem cell transcription factors Nanog and Oct4. These cells can differentiate into neurons, smooth muscle cells and melanocytes [125]. The hair follicle bulge area contains nestin-negative, K15-positive cells; these cells can differentiate into keratinocytes, neurons, glial cells and smooth muscle cells [126].

Human hair follicle stem cells promote nerve repair or the functional recovery of injured peripheral nerve and spinal cord [127].

**Figure 4:**
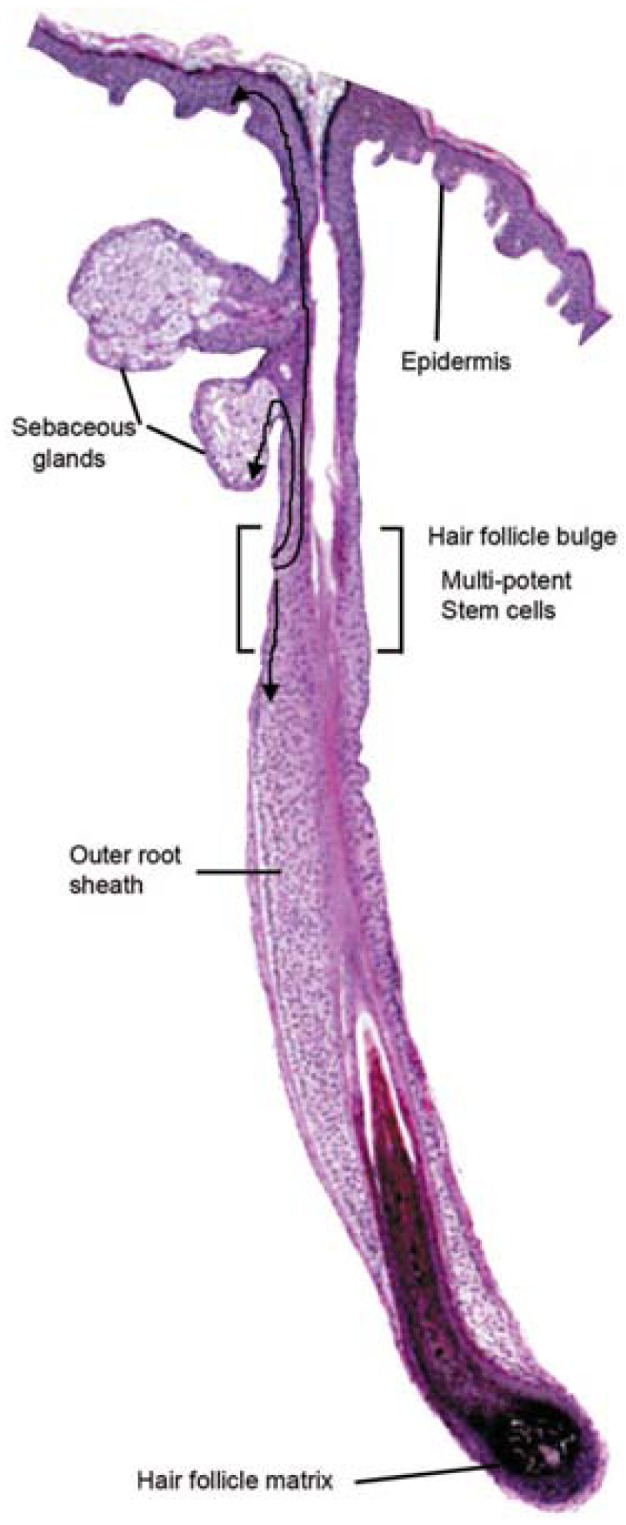
Hair follicle bulge and multipotent stem cells. Reproduced from [19] with permission from Wolters Kluwer.

Hair follicle bulge stem cells give rise to both hair follicle cells and epidermal cells. The hair follicle stem cells form epidermal stem cells only when the epidermis is wounded or stressed [128]. Bulge stem cells respond rapidly to epidermal wounding by generating short-lived TA cells responsible for acute wound repair [129] (Figure 4). Intense research is devoted to this promising source of stem-cell therapy to improve wound healing.

## 6. Directing Cell Fate for Regenerative Medicine

Regenerative medicine or cell-replacement therapy aims to treat human diseases caused by deficits in quality or quantity of particular cells, restoring damaged tissues in addition to alleviating the related symptoms. These diseases include neurodegenerative disorders, diabetes, liver and cardiovascular diseases, blindness, deafness, burns, and many others [130,131].

To accomplish its goal, regenerative medicine should be able to not only create these cells, but also to deliver them to patients. To create them, we can direct the cell fate of already available cells (ideally the patient's own cells, although age and comorbidities impair stem cell functionality [12,77]).

There are two main approaches to direct cell fate: (1) through directed differentiation, whereby cultured pluripotent stem cells (e.g., ESCs or iPSCs) follow several steps as they would *in vivo* or during embryonic development; or (2) via reprogramming or transdifferentiation, whereby a differentiated cell is converted directly into the cell of interest without proceeding through a pluripotent intermediate, most often by transcription factors [50,132-134].

Differentiation is performed *in vitro* by treating cells with recombinant growth factors (e.g., TGF-β superfamily, WNT and fibroblast growth factors, combined with co-culture systems), or with small molecules, which are homogenous, stable chemical compounds that are non-immunogenic and cheaper than proteins [135,136]. In addition, differentiation can be accomplished through spontaneously with embryoid bodies or floating clumps of cells [50].

Unfortunately, current protocols are still inefficient [137,138], but solutions for directed cell fate and transdifferentiation are under continuous investigation.

## 7. Stem Cells and Burns

Cell therapy has been used to treat burns since the introduction of composite epithelial autografts (CEA) by Green in 1975, evolving to dermal substitutes, later on to dermal-epidermal bio-engineered cultured skin substitutes and eventually to stem cells [139]. Stem cell therapy (allogeneic MSCs, iPSCs or immunomodulatory γδ TCells) after burn injury emerges as a promising treatment strategy, not only for wound healing, but also to treat systemic effects of burn trauma, the hypermetabolic response, inflammation (e.g., inflammatory-related diseases, such as acute lung injury/respiratory distress syndrome), and immunosuppression [5,140]. Stem cell therapy may offer an alternative to large volume resuscitation and be an adjunct to lung-protective ventilation strategies after severe burn injury [141]. Paracrine mechanisms and growth factor secretion, rather than post-engraftment differentiation and proliferation, seem to predominate in therapeutic effects of MSCs [71,139]. *In vivo*, MSCs attenuate proinflammatory cytokine release and nitric oxide production while upregulating the anti-inflammatory cytokines TGF-β, IL-10 and IL-12 [5]. MSCs also exhibit antiapoptotic, immunosuppressive and antifibrotic effects [62].

For the treatment of acute and chronic non-healing wounds (not burn related), combined gene delivery with stem cell therapy appears promising [12]. Gene therapy involves the insertion of a gene into recipient cells by viral transfection, naked DNA application, high pressure injection or liposomal vectors [142]. Sequential growth factor gene therapy delivers a cocktail of growth factor genes at strategic time points of wound healing [12,143].

To enhance the therapeutic response after stem cell treatment in burn patients, intense tissue engineering with the development of 3D scaffolds or matrices is of vital importance, as well as improved preconditioning cell treatments and optimized culture conditions [77].

### 7.1. The Challenge of Stem Cell Delivery in Burn Patients: A Link between Scaffolds and Wound Healing

Stem cell administration for burn patients still remains a challenge of intense research [140]. Intravenous infusion and local application of MSCs have been described in the clinical setting [144]. The concomitant use of acellular matrices or scaffolds is strongly recommended in order to increase cell homing, differentiation, mobilization and adhesion, eventually improving wound healing [144,145].

An ideal method for the effective administration of stem cells for burn patients has not yet been elucidated. Further comparison of the local and systemic effects in burn patients associated with each route of stem cell delivery needs to be performed. There is still not enough evidence in terms of analyzing systemic or local effects of stem cell delivery in burn patients, regarding different possible routes of administration. We still do not know exactly which percentage of locally delivered cells in a burn wound exerts its effects in wound healing and which, if any, affects the main circulation and has systemic effects, regarding the inflammatory and hypermetabolic response after a major burn. On the other hand, the efficiency of non-topical administration (such as intrapulmonary infused cells primarily to treat adult respiratory distress syndrome) for wound healing is unclear. To help clarify this, reporter gene imaging allows for stem cell tracking *in vivo*. Briefly, positron emission tomography is used to detect cell markers for assessment of the viability and location of stem cells after transplantation [146].

If we focus on wound healing, application of cells to the burn wound could be performed, either by the bedside as a non-invasive procedure, or in the operating room, immediately after debridement. The cells should be transferred on a matrix, scaffold or dermal substitute. One method is to first spray cells onto the wound with fibrin sealant [93] and afterwards cover with a dermal substitute, skin graft or film. The cover over the cells acts not only as a temporary dressing, but also to theoretically enhance cell paracrine signaling and homing of the cells, improving wound healing [147]. Although the use of cell administration using spray technologies is currently being performed in the clinical setting, there are no conclusive data to support its validity as a matrix vehicle.

Cutaneous wound healing requires the well-orchestrated integration of cell migration and proliferation, as well as extracellular matrix deposition, angiogenesis, and remodelling [12]. Delays in burn wound closure worsen a patient's susceptibility to infection, prolong pain, increase the total number of operative procedures, increase the incidence of hypertrophic scarring, and lengthen hospital stays. Stem cell therapies in wound care may lessen these morbidities [140]. Specifically, the burn wound has unique characteristics that have to be considered when designing a clinical trial for stem cell therapy applications: it is an ischemic wound, with an altered pH and temperature, prone to infection and development of chronic sequelae such as non-healing ulcers (sometimes even with malignant conversion) and hypertrophic scarring [148,149]. Furthermore, a major burn represents a handicap, with uncovered wounds open to air, which require frequent operations and dressing changes, and with long periods of immobilized hospital stay, which involve frequent position changes and physiotherapy, to avoid pressure sores, enhance rehabilitation and improve overall prognosis. This dynamic paradigm popularized the use of polymeric films for repair and closure of wounds. These films are semipermeable, microperforated and transparent materials that create an accelerated healing environment while avoiding dehydration, trauma and infection over the injury [150]. Additionally, radiofrequency applied to wound-contacting nano-engineered polymeric films (iron oxide-coated biodegradable nanoparticles and/or fibers -NPFs-) has been used to debride the wound. This may represent a novel burn treatment method, once stronger scientific evidence is available [151].

Several studies support the use of “intelligent matrices”, natural acellular matrices such as a porcine dermal acellular matrix accompanied by NPFs coated with monoclonal antibodies (e.g., anti-CD44) and loaded with specific growth factors, cytokines and antibiotics. The growth factors and cytokines improve the homing of autologous circulating MSCs, often elevated in patients with large burns [152], and also of delivered stem cells in general [144]. Chemoattractants for BM-MSC include hepatocyte growth factor, basic fibroblast growth factor and CXCL5, for instance [153]. Indeed, recruiting endogenous stem cells to the site of injury represents an alternative to transplantation; however, direct application of stem cells appears advantageous over diffusible growth factor administration. Stem cells can interact with their environment and release multiple wound healing factors [12]. Unfortunately, tissue-engineered dermal equivalents lack blood vessels and may act as possible barriers against nutrients necessary for keratinocyte or stem cell survival on top of such composites [144]. The time required to reperfuse the skin substitute increases the duration of ischemia, enhancing the risk of infection and graft destruction. One approach to minimize ischemia is through the use of BM-MSCs, which secrete VEGF [153]. On the other hand, VEGF could have detrimental effects in scarring [149]. Furthermore, in severe burn trauma with sepsis, bone marrow suppression has been described. Ichoka *et al.* investigated the effect of a BM-MSC impregnated collagen matrix on wound healing in a microcirculatory mouse model and observed significant increases in angiogenesis [153]. Other models of delayed wound healing in diabetes also report stem cells stimulating wound healing. Fiorina *et al.* showed that bone-marrow progenitor cells BM/PCs mobilize to the site of injury during diabetic wound repair in db/db mice, where increased levels of stem cells corresponded to higher levels of wound reepithelialization [154].

Ha *et al.* showed that transplantation of BMSCs transfected with an adenovirus hepatic growth factor gene can accelerate wound repair in diabetic rats [155]. Tark *et al.* showed that human CB-MSCs improved wound healing, when applied to diabetic mice, probably due to TGF-β [156].

Promising preclinical results with stem cells in wound healing are encouraging further research. In 2006, a patient bearing a diabetic foot was treated with a combination of autologous BM-MSCs and autologous fibroblasts on a biodegradable collagen membrane. Wound size decreased and vascularity increased [144]. Currently, nanoengineered multifunctional acellular biologic scaffolds, films and wound dressings emerge as delivery vehicles for stem cells in burn wounds. Although many types of tissue-regenerating stem cells may be used clinically, adding endometrial stem cells to MSCs may improve the vascularisation of these tissue engineered-constructs and significantly improve the outcomes of severely burned patients [144,145].

### 7.2. Legislation, Biosafety and Biotechnology Industry in Cell Therapy for Burn Care

Stem cell research is in its early stages of development and the market is therefore still immature. Approximately 1.1 million people affected by burns and other wounds in the U.S. would benefit from cell therapy products [157]. Although the preliminary results achieved to date raise great expectations, many pharmaceutical companies are reluctant to enter this market. To date, the most profitable strategy has been the signing of agreements between big pharmaceutical and other small biotechnology companies whose activity is entirely devoted to cell therapy and regenerative medicine. In addition, many stem cell-derived products are developed at universities and basic research institutions, where preclinical studies are also conducted.

Cell therapy, gene therapy and tissue engineering are considered ‘advanced therapy products’ (ATPs). As such, they should follow a regulatory framework to ensure patient accessibility and governmental assistance. An effective regulation implies scientific reality and objectivity, as well as flexibility to adapt as technology changes [159].

The U.S. Food and Drug Administration (F.D.A.) defines somatic cell therapy as the administration of autologous, allogeneic or xenogeneic non-germ cells excluding blood products for transfusion, which have been manipulated, processed, propagated, expanded, selected *ex vivo*, or drug-treated [160]. Cell therapy products are considered drugs, so they follow the same regulations, requiring a strict control of manipulation and facilities. Cell therapy products should adhere to the Current Good Manufacturing Practices, including quality control and quality assurance programs, which establish minimum quality requirements for management, staff, equipment, documentation, production, quality control, contracting-out, claims, product recall, and self-inspection [159]. The key points of the current FDA regulation for cell therapy products include: demonstrations of preclinical safety and efficacy; no risk for donors of transmission of infectious or genetic diseases; no risk for recipients of contamination or other adverse effects of cells or sample processing; specific and detailed determination of the type of cells forming the product and what are their exact purity and potency; *in vivo* safety and efficacy of the product [161]. Cell therapy products must be carefully described, stating whether autologous, allogeneic or xenogeneic cells are administered. According to the U.S. F.D.A., human cells are considered xenogeneic cells provided there has been any *ex vivo* contact with living non-human cells, tissues or organs [160]. It should also be detailed whether cells have been manipulated together with biomaterials, growth factors or serum, which is common in burn applications. Indeed, the biomaterial itself may have a more important role than the cell product [159]. Biomaterials for cell therapy should be biocompatible to prevent immune rejection or necrosis, biodegradable and assimilable without causing an inflammatory response, and have certain structural and mechanical properties [162]. Whether natural or artificial, biomaterial type and use is related to the route of administration of cell therapy protocols, implantation or injection. In the latter, which are simpler, biomaterials are usually in a hydrogel state, forming a hydrophilic polymer network, as occurs in polyethylene oxide, polyvinyl alcohol, polyacrylic acid, agarose, alginate, collagen and hyaluronic acid. Control of the biomaterials' porous structure is very important for increasing their efficacy in tissue regeneration [159,162].

Regarding the production process, a detailed description must be given of all procedures related to product quality in the Standard Operating Procedures, as for conventional medical products. The purity, safety, functionality and identity criteria used for conventional drugs must be met. Development of techniques for cell identification within a mixed cell culture population and for follow-up of transplanted cells will also be essential to ascertain the potential *in vivo* invasive processes and to ensure biosafety [159]. The production process must occur in a highly aseptic environment with comprehensive controls of both raw materials and handlers; it has to be reproducible and validated. Facilities where products are handled, packaged and stored should be designed and organized according to the Good Manufacturing Practice for Pharmaceutical Manufacturers (G.M.P.) guidelines [163]. Production and distribution should be controlled by the relevant local or national authorities based on the International Conference on Harmonization of Pharmaceuticals for Human Use, which standardized the potential interpretations and applications of the corresponding recommendations [164]. It is of paramount importance to prevent potential contamination, both microbiological and by endotoxins, due to defects in environmental conditions, handlers, culture containers or raw materials, or cross contamination with other products prepared at the same production plant. The number of technical staff should be the minimum required and should be trained in hygiene measures required for manipulation in a clean room [159].

In summary, aspects to be regulated mainly include control of development, manufacturing and quality using release and stability tests; non-clinical aspects such as the need for studies on biodistribution, cell viability and proliferation, differentiation levels and rates, and duration of *in vivo* function; and clinical aspects such as special dose characteristics, stratification risk and specific pharmacovigilance and traceability issues [159-161].

Since new stem cell-based therapies develop very fast, the regulatory framework must also adapt, although legislation may be expected to change more slowly.

## 8. Concluding Remarks

Stem cell therapy, regenerative medicine and tissue engineering emerge as innovative therapeutic strategies for a wide range of diseases, including burn injury. Stem cell therapy represents an interesting research field. Before we can offer this multidisciplinary promising treatment strategy clinically, preclinical studies are needed in order to satisfy safety concerns, improve efficiency of cell transplantation, and to design scaffolds or matrices by tissue engineering.

## Figures and Tables

iPSCs are generated from adult somatic cells through somatic cell nuclear transfer (SCNT), cell fusion or direct reprogramming, by ectopic expression of defined transcription factors. iPSCs are pluripotent stem cell lines suitable for disease modeling, autologous cell therapy, drug screening and basic research.

The umbilical cord contains vessels (two arteries and one vein), Wharton's jelly and the outer membrane.

Schematic representation of the cutaneous epithelium and the cell lineages derived from multi-potent stem cells in the hair follicle bulge. Under steady-state conditions, the stem cells are quiescent. At the beginning of the hair cycle when the lower follicle and matrix is regenerated, the stem cells in the bulge proliferate to give rise to new hair follicle keratinocytes. Under wounding conditions, the stem cells produce daughter cells that migrate from the niche to re-populate the basal layer of the epidermis, and the sebaceous gland.

**Table 1:** Main differences between epidermis and dermis skin layers.

**SKIN LAYERS**	**ORIGIN**	**VASCULARITY**	**CHARACTERISTICS**		**MAIN CELLS**
EPIDERMIS	Ectoderm	Avascular	Keratinized stratified epithelium		Melanocytes Keratinocytes
DERMIS	Mesoderm	Vascular	Contains extracellular matrix and skin appendages		Fibroblasts Adipocytes Macrophages
			Superficial = Papillary	Highly vascular Lax	
			Deep = Reticular	Less vascular Dense	

The epidermis contains no blood vessels, and is nourished by diffusion from the dermis. The main cell types that make up the epidermis are keratinocytes, melanocytes, Langerhans cells and Merkels cells. The dermis is basically composed of connective tissue and contains skin appendages.

**Table 2:** Types of cutaneous stem cells.

Epidermal		Dermal	Sebaceous	Hair follicle	Sweat glands	Melanocytes	MSC	Neural	Endothelial
Interfollicular	Hair Bulge								

Cutaneous stem cells include epidermal stem cells (interfollicular and bulge stem cells), dermal stem cells, sebaceous stem cells, hair follicle stem cells, sweat gland stem cells, melanocyte stem cells, mesenchymal stem cells, neural stem cells and endothelial stem cells. The more abundant skin stem cells are the epidermal hair bulge stem cells. Only a small fraction of stem cells, the interfollicular stem cells, reside in the basal layer of the interfollicular epidermis. These stem cells maintain adult skin homeostasis and hair regeneration, but they also participate in the repair of the epidermis after trauma.
